# *QuickStats*: Drug Overdose Death Rates* Among Persons Aged ≥15 Years, by Age Group and Urban-Rural Status^†^ — National Vital Statistics System, United States, 2020

**DOI:** 10.15585/mmwr.mm7147a3

**Published:** 2022-11-25

**Figure:**
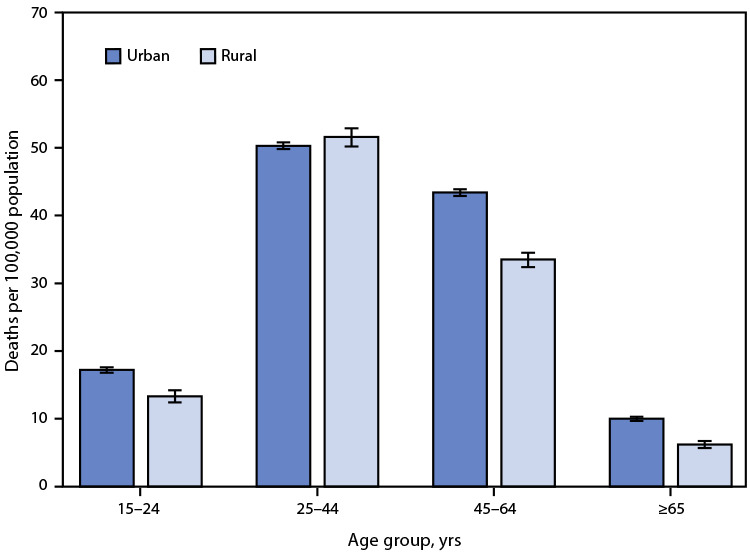
During 2020, death rates for drug overdose causes were higher in urban areas than in rural areas for those aged 15–24 years (17.2 compared with 13.3), 45–64 years (43.4 compared with 33.5), and ≥65 years (10.0 compared with 6.2). Among adults aged 25–44, drug overdose death rates were not significantly different between urban and rural areas (50.3 compared with 51.6). Drug overdose death rates were lower for adults aged ≥65 years compared with other age groups in both urban and rural areas.

**Source:** National Center for Health Statistics, National Vital Statistics System, Mortality Data, 2020 (https://www.cdc.gov/nchs/nvss/deaths.htm); CDC Wonder online database. https://wonder.cdc.gov/ucd-icd10.html

For more information on this topic, CDC recommends the following link: https://www.cdc.gov/drugoverdose/index.html

